# Cost-Effectiveness of Catheter Ablation for Rhythm Control of Atrial Fibrillation

**DOI:** 10.1155/2013/262809

**Published:** 2013-09-08

**Authors:** Gord Blackhouse, Nazila Assasi, Feng Xie, Kathryn Gaebel, Kaitryn Campbell, Jeff S. Healey, Daria O'Reilly, Ron Goeree

**Affiliations:** ^1^PATH Research Institute, McMaster University, Suite 2000, 25 Main Street West, Hamilton, ON, Canada L8P 1H1; ^2^Department of Clinical Epidemiology & Biostatistics, McMaster University, Hamilton, ON, Canada L8S 4L8; ^3^Centre for Evaluation of Medicines, St. Joseph's Healthcare, Hamilton, ON, Canada L8P 1H1; ^4^Population Health Research Institute, McMaster University, Hamilton, ON, Canada L8L 2X2

## Abstract

*Objective*. The objective of this study is to evaluate the cost-effectiveness of catheter ablation for rhythm control compared to antiarrhythmic drug (AAD) therapy in patients with atrial fibrillation (AF) who have previously failed on an AAD. *Methods*. An economic model was developed to compare (1) catheter ablation and (2) AAD (amiodarone 200 mg/day). At the end of the initial 12 month phase of the model, patients are classified as being in normal sinus rhythm or with AF, based on data from a meta-analysis. In the 5-year Markov phase of the model, patients are at risk of ischemic stroke each 3-month model cycle. *Results*. The model estimated that, compared to the AAD strategy, ablation had $8,539 higher costs, 0.033 fewer strokes, and 0.144 more QALYS over the 5-year time horizon. The incremental cost per QALY of ablation compared to AAD was estimated to be $59,194. The probability of ablation being cost-effective for willingness to pay thresholds of $50,000 and $100,000 was estimated to be 0.89 and 0.90, respectively. *Conclusion*. Based on current evidence, pulmonary vein ablation for treatment of AF is cost-effective if decision makers willingness to pay for a QALY is $59,194 or higher.

## 1. Background

Atrial fibrillation (AF) is the most common form of cardiac arrhythmia, associated with high morbidity and mortality. Based on the estimate of the Heart and Stroke Foundation, AF affects approximately 250,000 Canadians [1, 2]. This condition is characterized by disorganized, rapid, and irregular activity of the two upper chambers of the heart (atria), associated with irregular and rapid response of the two lower chambers of the heart (ventricles). Patients with AF are at higher risk of clot formation and subsequent adverse hemodynamic events such as stroke. AF increases the risk of stroke four- to five-fold across all age groups and is responsible for 10%–15% of all ischemic strokes [3]. The rate of hospitalization for AF in Canada was approximately 583 per 100,000 people, between 1997 and 2000, with an average of 129,000 hospitalizations per year [4].

AF may be classified on the basis of electrocardiographic findings or the frequency of episodes and the ability of an episode to convert back to sinus rhythm. AF is classified as a first-detected episode or a recurrent episode. Recurrent AF can be subclassified as paroxysmal (self-terminating, usually <24 hours), persistent (sustained >7 days), or permanent [5].

There are two main strategies for AF treatment: rhythm control (cardioversion and maintenance of sinus rhythm with antiarrhythmic drugs (AADs)) and rate control (atrio-ventricular (AV) nodal blockers and anticoagulation). The Canadian Cardiovascular Society (CCS) recommends both strategies as acceptable initial approaches. The only exception is for permanent AF, where rate control is recommended [6]. Various treatment options are available for rhythm control including medication, electrical (direct-current) cardioversion, or surgical procedures [7]. AAD therapy is recommended as a first choice for restoration of normal sinus rhythm (NSR) [7]. Three Class I drugs (flecainide, quinidine, and propafenone) and two Class III drugs (sotalol and amiodarone) are commonly used in Canada for treatment of AF [8]. Due to the limitations of these drugs in maintenance of NSR, along with side effects, nonpharmacological approaches including catheter ablation have been recently considered in treatment of AF [7, 9, 10]. The current standard surgical treatment is the Cox-Maze procedure, which requires open heart surgery [11, 12]. Because of the invasive nature of the Cox-Maze procedure, minimally invasive catheter-based interventions have been developed [13].

The goals of catheter ablation procedures are to eliminate triggers of AF and to modify the atrial substrate(s) responsible for the maintenance of AF [14]. Given that the pulmonary veins (PV) represent a critical anatomic site for the treatment of AF [15], minimally invasive procedures often involve the isolation of the source of abnormal impulses originating from these veins. In a minimally invasive catheter ablation procedure, a catheter is inserted through the femoral vein to access the heart and burn abnormal foci of electrical activity by direct contact or by isolating them from the rest of the cardiac atrium. Radiofrequency energy is most commonly used for AF ablation [16].

Antiarrhythmic drug therapy presents the advantage of being a noninvasive and commonly available therapeutic option but it may require chronic administration. Ablation of AF is associated with larger upfront costs but may be more successful in maintaining normal sinus rhythm over time. Therefore, there may be a trade-off between higher costs and better outcomes with AF ablation compared to antiarrhythmic medications. The objective of this paper is to assess the cost-effectiveness of treating paroxysmal AF patients with catheter ablation compared to antiarrhythmic drug treatment.

## 2. Methods

### 2.1. Overview

A Canadian specific cost-effectiveness analysis was conducted using a combined decision tree and Markov model for patients with AF. The treatment comparators in the model are (1) AF catheter ablation and (2) antiarrhythmic drug treatment (amiodarone 200 mg per day). The starting population are 65-year-old males with paroxysmal AF previously unsuccessfully treated with an AAD. Patients are assumed to have a CHADS_2_ [17] stroke risk score of 2. The CHADS_2_ index predicts the annual probability of stroke for individuals based on a risk score ranging from 0 to 6. This assumption is based on the mean CHADS_2_ score (2.1) amongst participants in the National Registry of Atrial Fibrillation [17]. The analysis was taken from the perspective of a publicly funded health care system. In the basecase analysis, the time horizon of the model was set to five years with a cycle length of three months. Although the clinical impact of AF treatment may go beyond five years, this time horizon was chosen due to the short-term nature (i.e., 12 months) of the randomized clinical trials comparing AF ablation with AAD. Alternate time horizons were tested in a sensitivity analysis.

### 2.2. Model Structure

**Figure 1:**
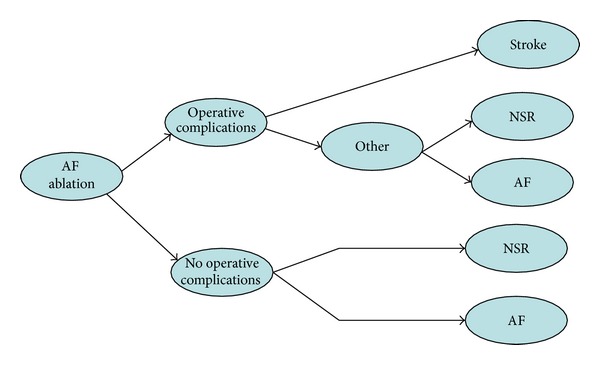
Structure of Short term model.

**Figure 2:**
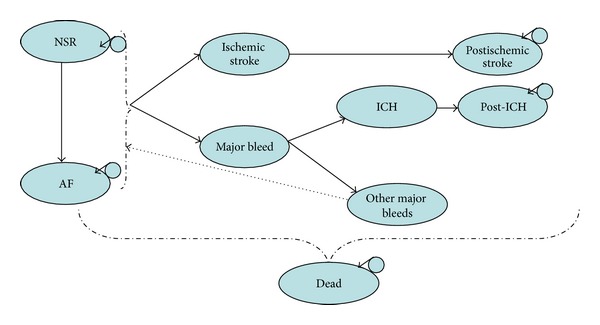
Structure of long-term model.

The model is comprised of both a one-year decision tree and a longer-term Markov model. Figures 1 and 2 graphically present the structure of the short-term and long-term models, respectively. As shown in Figure 1, a proportion of patients undergoing ablation are at risk of operative complications. These include cardiac tamponade, pulmonary vein stenosis, ischemic stroke, and transient ischemic attack. Patients suffering stroke are assumed to incur a permanent disability. Patients without an operative complication or with a nonstroke complication end the short-term model either in normal sinus rhythm (NSR) or with atrial fibrillation (AF). Patients in the AAD treatment group follow similar pathways as AF ablation patients during the one-year model. AAD patients are not at risk of operative complications but are at risk of pulmonary toxicity, which can be fatal, reversible, or irreversible. The proportion of patients in NSR after one year is based on freedom of AF outcomes from randomized controlled trials (RCTs) comparing AF ablation with AADs.

Figure 2 presents the structure of the long-term Markov model. Patients who are alive and not suffering a stroke after the one-year model enter the Markov model in either the NSR health state or the AF health state. In each three-month model cycle, patients are at risk of ischemic stroke and major bleeding events due to concomitant anticoagulants. The risk of stroke differs between patients in the NSR and AF health states. AF ablation patients are assumed to discontinue warfarin three months after their procedure, resulting in different bleeding risks between AF ablation patients and AAD treated patients. A proportion of major bleeds are intracranial haemorrhages (ICH). For simplicity, the remainder of the major bleeds is assumed to be gastrointestinal. 

Patients in the AF ablation treatment arm who do not achieve normal sinus rhythm after 1 year or have a subsequent recurrence of AF are assumed to switch to AAD treatment. Patients in the AAD treatment arm who do not achieve normal sinus rhythm after 1 year or have a subsequent recurrence of AF are assumed to remain on AAD treatment. There are separate health states to distinguish between the first and subsequent years after ischemic stroke and ICH. In every three-month cycle, patients in the NSR health state can revert back to the AF health state. Each health state is associated with different costs, utilities, and mortality rates.

### 2.3. Clinical Parameters

Various clinical model input parameters were used to populate the model. These parameters were used to estimate the expected QALYs and the expected number of strokes for each treatment group. These include the probability of achieving NSR for each treatment group; the probability of ischemic stroke; the probability of major bleeding; the probability of reverting to AF after achieving NSR; the probability of AF ablation procedural complications; the probability of pulmonary toxicity while being on an AAD; utility values for the various health states; mortality associated with the various health states. Data from a systematic review conducted as part of a health technology assessment on AF ablation was used to estimate the probability of normal sinus rhythm 12 months after ablation or antiarrhythmic treatment. Targeted reviews were undertaken to identify studies addressing the other clinical model variables. 

### 2.4. Normal Sinus Rhythm at 1 Year

The probability of being in normal sinus rhythm at one year was derived from data presented in a systematic review conducted by Assasi et al. [18]. In this systematic review, the authors identified five RCTs [19–23] that evaluated the proportion of patients in normal sinus rhythm 12 months after receiving either AF ablation or second-line ant-arrhythmic therapy. For the AAD treatment strategy, the probability of being in NSR after one year was estimated by pooling data from the AAD arms of these five RCTs using random effects meta-analysis [24]. The pooled probability of being in NSR at 12 months for the AAD treatment arm was estimated to be 0.26 (95% CI: 0.17, 0.34).  Table 1 provides the proportion of AAD in NSR at one year for each of these studies.

Assasi et al. [18] reported the relative risk of being in NSR at 12 months for AF ablation patients compared to AAD patients to be 2.93 (2.09, 4.11) in five RCTs of previous AAD failures. Based on this relative risk and the probability of being in NSR for the AAD treatment arm, the probability of AF ablation patients being in NSR at one year was estimated to be 0.756 (2.93 × 0.26). 

### 2.5. Probability of Ischemic Stroke

The annual probability of ischemic stroke for patients while in the AF health state was based on the CHADS_2_ classification system published by Gage et al. [17]. The probability of stroke by CHADS_2_ score [17] was estimated using a US registry of patients with AF. The CHADS_2_ stroke probabilities were applied to patients while being in the AF health state of the model. A CHADS_2_ risk score of 2, which corresponds to an annual risk of stroke 0.04, was assumed in the basecase analysis. In sensitivity analysis, the model is run assuming different risk scores.

A post hoc study [25] investigating the risk factors for ischemic strokes amongst patients enrolled in the AFFIRM trial [18] found that patients with AF had a 1.6 times greater risk of stroke than patients who were in normal sinus rhythm after treatment. Therefore, patients with normal sinus rhythm were assumed to have a relative risk reduction of stroke compared to patients with AF of 1/1.6, which is equal to 0.625. For example, if patients with AF are assumed to have a 0.04 annual risk of stroke, patients in normal sinus rhythm would be assumed to have an annual risk of stroke of 0.04 × 0.625, which is equal to 0.025. In sensitivity analysis, the model is run assuming that being in normal sinus rhythm has no impact on stroke risk. 

### 2.6. Major Bleeds

It is assumed that the proportion of patients taking warfarin in both treatment groups entering the model was 0.44 [26]. This was based on the percentage of patients in the FRACTAL registry that remained long term users of warfarin therapy after being diagnosed with atrial fibrillation [26]. The probability of bleeding while being on warfarin and aspirin therapy was based on data presented in a systematic review and meta-analysis published by Lip and Edwards [27]. First, the annual risk of major bleed while not receiving either warfarin or aspirin therapy was estimated based on details reported for the placebo arms of the studies included in the systematic review. These details included the number of patients and number of major bleeds in the placebo arm of each included study along with the mean duration of each study. Based on this data, the annual rate of a major bleed in the absence of warfarin or aspirin therapy was estimated to be 0.0058. 

Lip and Edwards [27] reported the relative risk of a major bleed for placebo compared to warfarin to be 0.45 (95% CI 0.25, 0.82). This relative risk was applied to the rate of major bleed without warfarin therapy to estimate the annual probability of major bleed while on being warfarin therapy. The annual probability of major bleed while being on warfarin therapy was estimated to be 0.0129 (0.00458 × 1/0.45). 

Data from the Stroke Prevention in Atrial Fibrillation II Study [28, 29] were used to estimate the proportion of major bleeds that are ICHs. The annual rate of major haemorrhages along with the annual rate of ICH for patients on aspirin and warfarin therapy was presented. Based on this data, the proportion of major bleeds that are ICHs was estimated to be 0.332. 

### 2.7. Recurrence of Atrial Fibrillation

The probability of recurrence of AF after achieving NSR was derived from a long-term observational study of AF ablation and AAD treatment by Pappone et al. [30] who reported the probability of being free of AF at one, two, and three years for both AAD and ablation treated patients. Based on the one- and three-year data, the annual probability of AF recurrence for patients achieving NSR was estimated to be 0.036 for AF ablation treated patients and 0.221 for AAD treated.

### 2.8. Procedural Complications and Adverse Events

The probability of AF ablation procedural complications was taken from a systematic review of RCT and non-RCT studies evaluating catheter-based AF ablation procedures [31]. The probabilities of ischemic stroke, transient ischemic attack, cardiac tamponade, and pulmonary veins stenosis resulting from AF ablation are 0.003, 0.002, 0.008, and 0.016, respectively. 

The probability of pulmonary toxicity while being on AAD treatment was based on data presented by Vorperian et al. [32]. Vorperian et al. [32] performed a meta-analysis for adverse events while being on low dose amiodarone. The authors report that 1.9% of amiodarone patients from their four included studies suffered from pulmonary toxicity. Based on the weighted mean followup in the studies (27.54 moths), the annual probability of pulmonary toxicity was estimated to be 0.00832. The proportion of cases of pulmonary toxicity that are irreversible was assumed to be 0.25 [33]. The probability of death following pulmonary toxicity was assumed to be 0.091 as reported by Dusman et al. [34].

### 2.9. Mortality

General population age and gender specific mortality rates based on Canadian Life Tables [35, 36] were applied to patients in the model in the absence of events (e.g., ischemic stroke and major bleeds). Several sources were used to derive mortality rates for patients who suffer an ischemic stroke during the model. Johansen et al. [37] reported various outcomes for 34,448 patients hospitalized for first stroke in Canada. Amongst the outcomes reported were 28-day mortality rates by age group and gender according to stroke type. The 28-day mortality rates for patients with strokes classified as cerebral infraction (ICD-9 434,436) were applied to patients suffering an ischemic stroke in the model. To account for the increased risk of death following the remainder of the first year after stroke, data from another Canadian-based publication was used. Tu and Gong [38] reported mortality for patients hospitalized for acute stroke in Canada at 30 days (18.9%) and at one year (32.0%). Data was not presented by stroke type or by age or gender. The ratio of one-year mortality to 30-day mortality reported in this study can be estimated as 1.78 (0.32/0.189). In the model, this factor was applied to the 28 mortality rates reported by Johansen et al. [37] in order to estimate one-year age and gender specific mortality rates for ischemic stroke. For poststroke mortality after one year, general population mortality was increased by a factor of 2.3. This was based on a long-term population-based stroke study [39]. Mortality after ICH was estimated in a similar manner. Thirty-day age and gender specific mortality after intracranial hemorrhage (ICH) (ICD9-431) was taken from the Canadian-based study by Johansen et al. [37]. To estimate one-year mortality, the 30-day mortality rates were increased by a factor of 1.2. This adjustment factor was based on a study on long-term mortality post-ICH [40]. Flaherty et al. [40, 41] reported mortality to be 0.48 and 0.59 at one month and one year, respectively. 

### 2.10. Utilities

While being in the normal sinus rhythm health state, patients were assigned age and gender specific general population utility values [42]. No existing published studies primarily reporting utilities for AF were identified. However, Reynolds et al. [43] do describe various utility estimates they derived as part of their cost-effectiveness analysis of the radio frequency AF ablation. Reynolds et al. [43] specifically transformed patient level SF-12 responses for patients enrolled in the FRACTAL registry to utility scores using the Brazier algorithm [44]. The FRACTAL registry included over 1000 patients with a first time diagnosis of AF. Reynolds et al. [43] reported the average change in utility in patients with no documented recurrences of AF over 12 months to be 0.046. Based on this data, a disutility of 0.046 was applied to patients while being in the AF health state. 

It is worth noting that Reynolds et al. estimated utilities transforming SF-36 patient level responses from patients before and after AF ablation from two other patient populations. However, we felt the data from the FRACTAL trial to best represent the change in utility moving from an AF to NSR health state, as the other studies evaluated intervention specific changes in utility values.

Utility weights were estimated for both ischemic and haemorrhagic stroke. Data from two studies were used to derive stroke health state utility weights. Riviero-Arias et al. [45] provided estimates of poststroke utility scores according to modified Rankin Score (mRS). In a Canadian-based cohort study, Goeree et al. [46] reported the distribution of discharge modified ranking score according to the type of stroke (ischemic, haemorrhagic, and transient ischemic attack). The mRS specific utility values reported by Riviero-Arias et al. [45] were applied to the distribution of hospital discharge mRS reported by Goeree et al. in order to derive a weighted average utility weight for ischemic and haemorrhagic stroke. Based on this data, the utility weight applied to patients after ischemic and haemorrhagic stroke was 0.46 and 0.28, respectively. 

A disutility of 1.0 for seven days was applied to the AF ablation complications. For pulmonary toxicity, a disutility of 1.0 for the duration of a related hospitalization was applied. The mean length of stay for a pulmonary toxicity related hospitalization was estimated to be 13 days. This was based on hospitalizations identified from the Ontario Case Costing Initiative database [47] with a primary diagnosis of J70.2 (acute-drug induced interstitial lung disorders), J70.3 (chronic drug-induced interstitial lung disorders), J70.4 (respiratory conditions due to drug-induced interstitial lung disorders, unspecified), or J84.1 (idiopathic pulmonary fibrosis). For irreversible pulmonary toxicity, a utility weight of 0.6 was applied to each cycle [48].

### 2.11. Cost of Ablation Procedure

The cost of an entire inpatient stay for an ablation procedure was estimated using data from the Ontario Case Costing project [47]. We specifically used the average cost of hospitalizations with a primary procedure code indicating catheter ablation using a percutaneous transluminal approach (1.HH.59.GP-AW) and the most responsible diagnosis of atrial fibrillation (I480). Based on these criteria, the mean cost per AF ablation hospitalization was estimated to be $7,056. The physician fees for an AF ablation procedure were estimated using expert opinion on applicable physician fee codes and fees listed in the Ontario schedule of benefits for physician services [49]. The total physician fees for an AF ablation procedure were estimated to be $2,534 The total cost per AF ablation used in the model was $9,590. The number of ablation procedures per patient was derived from a survey of electrophysiology laboratories published by Cappato et al. [50]. Cappato et al. reported that amongst 8745 AF ablation patients, 2389 (27%) required one or more ablation procedures. Therefore, in the model it was assumed that each patient would require 1.27 ablation procedures and the total procedure costs applied to the AF-ablation arm were $12,179 ($9,590 × 1.27). In the first year after ablation, patients were assumed to be followed up with three cardiologist consultations and undergo a CT scan during the first year after ablation. The cumulative cost of followup in the first-year follow-up was $666. No follow-up costs were applied after the first year after ablation.

### 2.12. Procedural Complication Costs

The cost of procedural complications was estimated from a published Canadian-based costing study by Khaykin et al. [51]. The unit costs of cardiac tamponade, PV stenosis, stroke, and transient ischemic attack specifically were $5,842, $8,487, $14,1872, and $4.297, respectively. These costs included inflation to 2010 $CDN using the healthcare component of the consumer price index. The acute cost of pulmonary toxicity was estimated from the Ontario Case Costing Initiative. The mean cost per hospitalization with a primary diagnosis potentially related to pulmonary toxicity was found to be $20,436. These were hospitalizations with a primary diagnosis code of J70.2 (acute drug-induced interstitial lung disorders), J70.3 (chronic drug-induced interstitial lung disorders), J70.4 (respiratory conditions due to drug-induced interstitial lung disorders, unspecified), or J84.1 (idiopathic pulmonary fibrosis). For irreversible pulmonary toxicity, an annual cost of $3,799 was applied [52]. These costs included inflation to 2010 $CDN using the healthcare component of the consumer price index [53].

### 2.13. Medication Costs

Unit costs for medications were based on reimbursement prices from the Ontario Drug Benefit Formulary [54]. The assumed daily dose of amiodarone was 200 mg per day. An 8% pharmacy markup was applied to the raw drug costs along with a $7.00 dispensing fee. It was assumed that a 90-day supply of the drug would be dispensed each time. The total annual cost of amiodarone was estimated to be $433.29. Both patients with normal sinus rhythm and those in the AF health state were assigned amiodarone costs each cycle.

The assumed daily dose of warfarin was 5 mg per day. An 8% pharmacy markup was applied to the raw drug costs along with a $7.00 dispensing fee. It was assumed that a 90-day supply of the drug would be dispensed each time. The total annual warfarin cost used in the model was $75.30. In addition, an annual monitoring cost for warfarin of $387.54 was assumed in the model [55]. This cost included inflation to 2010 $CDN using the healthcare component of the consumer price index [40, 53].

### 2.14. Stroke and Major Bleed Costs

The cost of stroke was estimated from a Canadian-based costing study by Goeree et al. [46]. In this study, the total one year health care cost for patients suffering an ischemic stroke was found to be $53,576. The one year cost following a haemorrhagic stroke was estimated to be $56,573. These costs were applied to the first year after stroke in the current model. Goeree et al. presented stroke costs by different resource categories. Costs for the second year and after stroke were assumed to be equal to the sum of the costs for long-term care, home care, prescription medications, outpatient visits, doctor visits, and assistive devices. These costs amounted to $6,265 and $4,841 for ischemic and haemorrhagic stroke, respectively. After inflating to 2010 $CDN [53], the first-year costs for ischemic and haemorrhagic were $61,413 and $58,159, respectively. Each subsequent year's costs for ischemic and haemorrhagic stroke were $6,801 and $5,843, respectively. The cost of a major bleed used in the model was $6,023 and was based on the average cost of a hospitalization with a case mix group for a gastrointestinal bleed (CMG 281).

### 2.15. Variability and Uncertainty

Parameter uncertainty is evaluated using probabilistic sensitivity analysis (PSA) and is presented in the form of a cost-effectiveness acceptability curve for the AF ablation strategy. One thousand Monte Carlo simulations were used in the PSA.  Table 2 presents the distributions, parameters, and resultant 95% confidence intervals for model parameters. 

Model structural uncertainty was assessed using one-way sensitivity analysis. Cost-effectiveness specifically results presented are varying discount rates, model time horizon, the annual probability of AF recurrence after ablation, and the disutility of being in the AF health state. Additionally, the model was run under the assumption that restoration of NSR does not impact the stroke risk and under the assumption that amiodarone treatment is discontinued for patients not in normal sinus rhythm.

Variability of different patient groups was also assessed using one way sensitivity analyses. Model results according to age and gender were tested along with model results by baseline CHADS_2_ stroke risk score. Sensitivity analysis on age and gender was conducted under two scenarios. In the first scenario, the same probability of stroke was assumed for all age groups. The probability of stroke was the same for all ages given a specific CHADS_2_ score. In the second scenario, the risk of stroke is varied according to age using Framingham stroke risk data [56]. Wolf et al. [56] presented the 10-year risk of stroke according to age and gender. Using this data, the relative risk for stroke for different ages relative to 65 year olds was estimated and applied to the risk of stroke based on a CHADS_2_ risk score of 2. 

## 3. Basecase Results

Table 3 presents the cost-effectiveness results for the basecase analysis. As shown, the ablation treatment group was estimated to incur $8,539 in incremental expected costs compared to the AAD treatment group. The model estimated that the ablation group had more expected QALYs and fewer expected number of strokes compared to the AAD treatment group. The ablation treatment arm specifically was estimated to result in 0.033 fewer expected number of strokes and 0.144 more quality adjusted life years compared to the AAD treatment arm. Based on the expected costs and QALYs estimated by the model, the incremental cost per QALY of the ablation arm compared to the AAD arm is $59,194. Therefore, using the basecase analysis, ablation would be considered cost-effective if societies' willingness to pay for a QALY is $59,194 or higher. Otherwise, AAD therapy is the cost-effective treatment strategy. 

**Figure 3:**
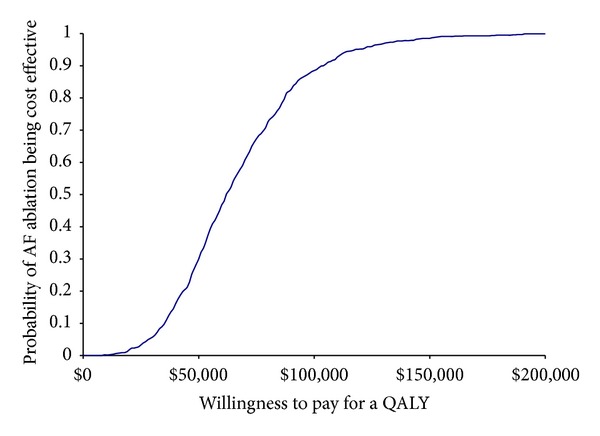
Cost-effectiveness acceptability curve.

Figure 3 presents the cost-effectiveness acceptability curve for the AF ablation strategy compared to the AAD strategy. AF ablation has the highest probability of being cost-effective at willingness to pay values of $62,000 per QALY and higher. The probability of AF ablation being cost-effective at willingness to pay for a QALY threshold of $25,000, $50,000, $100,000, and $150,000 was estimated to be 0.03, 0.30, 0.89, and 0.98, respectively.

## 4. One-Way Sensitivity Analysis 

Table 4 presents cost-effectiveness results for different patient groups based on age, gender, and CHADS_2_ index score. As shown, cost-effectiveness results varied according to age and gender. The incremental cost per QALY is estimated to be $57,088 for a 55-year-old female and $65,129 for 75-year-old female. For males, the incremental cost per QALY was estimated to be $57,167 for a 55 year old and $65,129 for a 75 year old. Cost-effectiveness results did vary according to the CHADS_2_ index score. When the CHADS_2_ index score for the model cohort is 0, the incremental cost per QALY of ablation compared to AAD becomes $68,822. When the CHADS_2_ index score was 4, the incremental cost per QALY became $44,652. The time horizon chosen had a large impact on results. Using a 10-year time horizon, the incremental cost per QALY became $14,273 for AF ablation compared to AAD. A 20-year time horizon results in ablation being both less costly and more effective than the AAD treatment strategy. Discount rates had a relatively small impact on results. If a zero percent discount rate is used, the incremental cost per QALY of the ablation strategy became $49,308 per QALY. In their economic evaluation of ablation compared to AAD, Reynolds et al. assumed that restoration of NSR had no impact on the risk of stroke. If this same assumption is applied in the current model, the cost per QALY of the AF ablation strategy became $86,129. As shown, even with the assumption that restoration of NSR does not impact stroke risk, the incremental QALYs from AF ablation were only reduced from 0.144 in the basecase to 0.116. This indicates that much of the difference in QALYs predicated by the model was due to the disutility applied to patients while in the AF health state. If the disutility of being in AF compared to NSR is 0.08 instead of the basecase value of 0.043, the cost per QALY of AF ablation becomes $38,390 per QALY. If the disutility of being in the AF health state is 0.02 or 0.00 the incremental cost per QALY becomes $101,083 per QALY and $221,839 per QALY, respectively. The annual probability of recurrence of AF in patients undergoing ablation has little impact on the cost per QALY estimate. If the annual probability of AF recurrence is assumed to be 0.00, the cost per QALY becomes $53,831. If the annual probability of AF recurrence is assumed to be 0.05, the cost per QALY becomes $61,280. 

## 5. Discussion

The current study found the incremental cost per QALY of catheter ablation of AF to be $59,194 in Canada compared to treatment with amiodarone in patients previously failing an antiarrhythmic medication. Based on common cost-effectiveness thresholds, this may be considered to be a cost-effective strategy. Our findings are similar to those from economic evaluations of AF ablation conducted from the perspective of other countries. McKenna et al. [57] evaluated the cost-effectiveness of AF ablation compared to treatment with Amiodarone from a UK perspective. The authors assumed a lifelong time horizon in their basecase and estimated the cost-effectiveness of AF ablation to be *£*7,910 per QALY for patients with a CHADS risk score of 3. Assuming a 5-year time horizon, the cost-effectiveness of AF ablation in this patient group was estimated to be *£*20,831 ($Can32,704). Reynolds et al. [43] conducted a US- based economic evaluation of catheter ablation for AF compared to treatment with Amiodarone in the same population as our study. Using a 5-year time horizon theoretical basecase, the authors estimated the cost per QALY of ablation compared to amiodarone to be $51,131. It is worth noting that Reynolds et al. [43] assumed no benefit of ablation on the subsequent risk of stroke. Therefore, the increase in QALYs was primarily due to better quality of life after ablation. Our study is unique because it is the first to look at the cost-effectiveness of catheter ablation from a Canadian perspective.

There are a number of limitations to this study. One limitation is that the published RCT comparing the proportion of patients achieving normal sinus rhythm after ablation or antiarrhythmic drugs is limited to 1 year followup. Assumptions had to be made on the effectiveness of the two strategies on maintaining rhythm control beyond the first year. Additionally, no published data was found directly comparing stroke rates between patients treated with ablation compared to those treated with AAD. 

## Figures and Tables

**Table 1:** Studies used to estimate probability of being in NSR at 1 year for AAD.

Study	*n*	*n* NSR at 12 months	Proportion NSR at 12 months	Weight of study
Forleo et al. (2009) [19]	35	15	0.43	15.5%
Jaïs et al. (2008) [20]	55	13	0.24	22.4%
Pappone et al. (2006) [22]	99	22	0.22	27.6%
Krittayaphong et al. (2003) [21]	15	6	0.40	8.8%
Wilber et al. (2010) [23]	61	10	0.16	25.7%

Pooled 0.26 (95% C.I. 0.17, 0.34).

**Table 2:** Distributions used for model parameters in probabilistic sensitivity analysis.

Variable	Distribution (parameters)	Mean	95% C.I. based on distribution and parameters
Normal sinus rhythm (NSR) variables			
Probability of remaining in normal sinus rhythm at 1 year for AAD	Beta (*α* = 26.47, *β* = 176.11)	0.26	(0.17, 0.34)
RR of NSR at 1 year with ablation	log normal (exp⁡(*μ* = 1.07, s.e. = 0.18))	2.93	(2.09, 4.11)
Probability (annual) of AF recurrence after NSR with AAD	Beta (*α* = 20.71, *β* = 550.23)	0.036	(0.00324, 0.00904)
Probability (annual) of AF recurrence after NSR with ablation	Beta (*α* = 122.53, *β* = 431.47)	0.221	(0.023, 0.053)
Disutility due to AF	Beta (4.6, 95.4)	0.046	(0.014, 0.095)

Treatment cost variables			
Annual cost of Amiodarone	Fixed	$433	
Cost of AF ablation hospitalization	Gamma (25, 282.24)	$7,056	($4566, $10,709)
AF ablation physician fees	Fixed	$2534	
Number of AF ablation procedures	Fixed	1.27	
Follow-up cost 1st year after ablation	Fixed	$666	

Perioperative ablation complication variables			
Probability of stroke as complication of AF ablation	Beta (*α* = 17, *β* = 5648)	0.003	(0.0017, 0.0046)
Probability of TIA as complication of AF ablation	Beta (*α* = 13, *β* = 5454)	0.002	(0.0012, 0.0038)
Probability of cardiac tamponade as complication of AF ablation	Beta (*α* = 45, *β* = 5678)	0.008	(0.0057, 0.0010)
Probability of PV stenosis as complication of AF ablation	Beta (*α* = 91, *β* = 5740)	0.016	(0.0017, 0.0049)
Cost of stroke as complication from AF ablation	Gamma (*α* = 25, *β* = 594.88)	$14872	($9624, $21243)
Cost of TIA as complication from AF ablation	Gamma (*α* = 25, *β* = 171.87)	$4296	($2,781, $6137)
Cost of cardiac tamponade as complication from AF ablation	Gamma (*α* = 25, *β* = 233.69)	$5842	($3781, $8345)
Cost of PV stenosis as complication from AF ablation	Gamma (*α* = 25, *β* = 339.47)	$8487	($5492, $12123)

Pulmonary toxicity variables			
Annual probability of pulmonary toxicity	Beta (6.14, 731.86)	0.008	(0.003, 0.016)
Probability of death from pulmonary toxicity	Beta (3, 30)	0.091	(0.019, 0.208)
Probability that pulmonary toxicity is irreversible	Beta (25, 75)	0.25	(0.171, 0.338
Cost of acute pulmonary toxicity	Gamma (25, 897.36)	$22434	($14518, $32044)
Annual cost of irreversible pulmonary toxicity	Gamma (*α* = 25, *β* = 151.98)	$3799	($2459, $5427)
Utility weight for irreversible pulmonary toxicity	Beta (60, 40)	0.6	(0.503, 0.693)

Ischemic stroke variables			
Annual probability of stroke (Chads_2_ = 2)	Beta (*α* = 58.97, *β* = 1415.21)	0.04	(0.031, 0.051)
Increase in risk of stroke in the presence of AF	log normal (exp⁡(*μ* = 0.47, s.e. = 0.18))	1.6	(1.11, 2.30)
First year cost of ischemic stroke	Gamma (*α* = 25, *β* = 2326.39)	$58159	($37638, $83076)
Subsequent years of ischemic stroke	Gamma (*α* = 25, *β* = 272.2)	$6801	($4401, $9715)
Utility weight for ischemic stroke	Beta (91.19, 108.80)	0.46	(0.39, 0.53)

Anticoagulation variables			
Proportion of patients on warfarin	Beta (442.2, 562.8)	0.44	(0.408, 0.471)
Annual cost of warfarin treatment and monitoring	Fixed	$463	
Annual probability of major bleed while being on placebo	Beta (15, 2579.6)	0.0058	(0.00324, 0.00904)
Relative risk of major bleed warfarin relative to placebo	log normal (exp⁡(*μ* = −0.798, s.e. = 0.306))	0.45	(0.25, 0.82)
Proportion of major bleeds that are ICH	Beta (966.96, 1942.04)	0.33	(0.315, 0.350)
First year cost of heamorrhagic stroke	Gamma (*α* = 25, *β* = 2456.53)	$61413	($39743, $87723)
Cost for subsequent years of hemorrhagic stroke	Gamma (*α* = 25, *β* = 210.10)	$5255	($3401, $7507)
Utility weight for hemorrhagic stroke	Beta (55.38, 144.62)	0.28	(0.22, 0.34)

**Table 3:** Basecase cost-effectiveness results.

Treatment	Expected costs	Expected strokes	Expected QALYs	$/QALY
Ablation	$21,150	0.122	3.416	
AAD	$12,611	0.155	3.272	
Incremental (Ablation-AAD)	$8,539	(0.033)	0.144	$59,194

**Table 4:** One-way sensitivity analyses.

	Incremental costs	Incremental QALYs	Incremental $/QALY
Sensitivity analysis by age and gender assuming the same risk of stroke for all starting ages			

Males			
Age			
55	$8,330	0.146	$57,167
60	$8,365	0.145	$57,846
65	$8,539	0.144	$59,194
70	$8,630	0.141	$61,120
75	$8,787	0.135	$65,129
Females			
Age			
55	$8,330	0.146	$57,088
60	$8,365	0.145	$57,765
65	$8,548	0.144	$59,219
70	$8,637	0.141	$61,142
75	$8,793	0.135	$65,147
CHADS_2_ risk score			
0	$9,259	0.135	$68,822
1	$8,941	0.139	$64,412
2	$8,539	0.144	$59,194
3	$7,952	0.152	$52,214
4	$7,242	0.162	$44,652
Time horizon			
3 years	$10,670	0.082	$130,711
5 years	$8,539	0.144	$59,194
10 years	$4,299	0.301	$14,273
20 years	($71)	0.505	AF ablation dominates
Discount rate			
0%	$7,995	0.162	$49,308
3%	$8,335	0.151	$55,211
5%	$8,539	0.144	$59,194
Restoring NSR has no impact on stroke risk	$10,019	0.116	$86,129
Disutility of AF health state			
0	$8,539	0.038	$221,839
0.02	$8,539	0.084	$101,083
0.04	$8,539	0.130	$65,454
0.06	$8,539	0.176	$48,396
0.08	$8,539	0.222	$38,390
Annual probability of recurrence of AF after ablation			
0.00	$8,230	0.153	$53,831
0.01	$8,317	0.150	$55,276
0.02	$8,403	0.148	$56,743
0.03	$8,486	0.146	$58,233
0.04	$8,569	0.143	$59,745
0.05	$8,650	0.141	$61,280
